# Mouse embryonic palatal mesenchymal cells maintain stemness through the PTEN-Akt-mTOR autophagic pathway

**DOI:** 10.1186/s13287-019-1340-8

**Published:** 2019-07-29

**Authors:** Lungang Shi, Binchen Li, Binna Zhang, Congyuan Zhen, Jianda Zhou, Shijie Tang

**Affiliations:** 1 Department of Plastic Surgery and Burn Center, the Second Affiliated Hospital of Shantou University Medical College, North Dongxia Road, Shantou, 515041 Guangdong China; 2 Shantou University Medical College, No. 22 Xinling road, Shantou, 515041 Guangdong China; 3 Center for Translational Medicine, the Second Affiliated Hospital of Shantou University Medical College, North Dongxia Road, Shantou, 515041 Guangdong China; 4 Department of Plastic Surgery, Third Xiangya Hospital, Central South University, Changsha, 410013 Hunan China

**Keywords:** Autophagy, Stemness, Mouse embryonic palatal mesenchyme cells, PTEN-Akt-mTOR signaling pathway

## Abstract

**Background:**

Both genetic and environmental factors are implicated in the pathogenesis of cleft palate. However, the molecular and cellular mechanisms that regulate the development of palatal shelves, which are composed of mesenchymal cells, have not yet been fully elucidated. This study aimed to determine the stemness and multilineage differentiation potential of mouse embryonic palatal mesenchyme (MEPM) cells in palatal shelves and to explore the underlying regulatory mechanism associated with cleft palate formation.

**Methods:**

Palatal shelves excised from mice models were cultured in vitro to ascertain whether MEPM are stem cells through immunofluorescence and flow cytometry. The osteogenic, adipogenic, and chondrogenic differentiation potential of MEPM cells were also determined to characterize MEPM stemness. In addition, the role of the PTEN-Akt-mTOR autophagic pathway was investigated using quantitative RT-PCR, Western blotting, and transmission electron microscopy.

**Results:**

MEPM cells in culture exhibited cell surface marker expression profiles similar to that of mouse bone marrow stem cells and exhibited positive staining for vimentin (mesodermal marker), nestin (ectodermal marker), PDGFRα, Efnb1, Osr2, and Meox2 (MEPM cells markers). In addition, exposure to PDGFA stimulated chemotaxis of MEPM cells. MEPM cells exhibited stronger potential for osteogenic differentiation as compared to that for adipogenic and chondrogenic differentiation. Undifferentiated MEPM cells displayed a high concentration of autophagosomes, which disappeared after differentiation (at passage four), indicating the involvement of PTEN-Akt-mTOR signaling.

**Conclusions:**

Our findings suggest that MEPM cells are ectomesenchymal stem cells with a strong osteogenic differentiation potential and that maintenance of their stemness via PTEN/AKT/mTOR autophagic signaling prevents cleft palate development.

## Background

The palate plays a vital role in shaping the embryonic facial primordia. Palate development in humans includes the primary palate formation (a small part of the adult hard palate) and secondary palate formation (hard and soft parts of the palate) [1]. Cleft palate is caused by abnormal formation of the secondary palate during embryonic development [2, 3]. It is one of the most common congenital birth defects (global incidence rate: 1 in 700 people). During normal palate development, the palatal shelves grow down vertically on both sides of the tongue till embryonic day 13.5 (E13.5); subsequently, these begin to elevate above the tongue at E14 and start growing horizontally towards each other. The initial contact of the palatal shelves initiates the formation of the medial edge epithelial (MEE) seam on E14.5; disintegration of the seam enables palatal fusion by E15.5 [4]. Since normal palate primarily comprises of mesenchymal cells surrounded by a thin layer of epithelial cells [2, 5–7], loss of viability of mouse embryonic palatal mesenchyme (MEPM) cells or disruption of extracellular matrix secretion by MEPM cells may result in cleft palate formation [8].

The majority of MEPM cells (> 90%) are derived from cranial neural crest cells (CNCCs) [9, 10]. Some studies suggested that the balance between MEPM proliferation and apoptosis may determine the size of palatal shelves and that reduction in the number of MEPM cells may delay or impair the embryonal development of palate [7, 11–15]. However, other studies have found no association between cell death and palatal mesenchyme development except in the vicinity of the medial epithelial seam (MES) [16, 17]. However, these studies did not involve in-depth characterization of the stemness and other characteristics of MEPM cells that may potentially influence cleft palate formation. The lack of a consensus in this respect suggests the existence of alternative mechanisms (such as the dysregulation of cellular events) during palate development that are linked to increased MEPM cell proliferation [18, 19]. Stem cells exhibit strong self-renewal and proliferation capacities and differentiation potential; initiation of differentiation tends to suppress proliferation. Hence, investigation of the mechanism that regulate stemness can clarify the identity of MEPM as stem cells and provide clearer insights into the mechanism of cleft palate development. In this study, we investigated the multilineage differentiation potential of MEPM cells to determine their stemness and also examined the underlying mechanisms that regulate palate development.

Autophagy is a dynamic catabolic process regulated by environmental and hormonal cues. It promotes the survival of stem cells under harsh conditions and can regulate embryonic development and differentiation [20–23]. Moreover, autophagic regulation of stem cell survival was shown to be associated with stemness [21–23] as stronger stemness showed a correlation with greater tolerance towards hypoxia [24]. Hence, we investigated the association between MEPM stemness and autophagy using mice models that are known to exhibit morphological and molecular similarities to humans with respect to palate development [25]. Specifically, we examined the contribution of the PTEN-Akt-mTOR autophagy signaling pathway to stemness of MEPM cells. Our findings may help characterize the true nature of MEPM and clarify the underlying mechanisms that determine its stemness and cleft palate formation.

## Materials and methods

### Animals

All experiments involving animals were approved by the Laboratory Animal Ethical Committee of the Medical College of the Shantou University, China. Fifty Kunming mice (15 males and 35 females; age 8–10 weeks) were purchased from the Vital River Laboratory Animal Technology Co. Ltd. (Beijing, China). Female mice were mated with fertile males overnight and the presence of a vaginal plug on the following morning was considered indicative of embryonic day 0.5 (E0.5). Pregnant females at E10.5 were randomly divided into two oral gavage groups: retinoic acid (70 mg/kg) (RA; Sigma, St. Louis, MO, USA) dissolved in sesame oil group, and 10 mL/kg sesame oil only control group.

### Cell culture

Palatal shelves (1 mm^3^ cubes) were excised from mice on E15 and plated as previously described [26] in Dulbecco’s modified Eagle’s medium (DMEM) containing 10% fetal bovine serum (FBS; Gibco, Grand Island, NY, USA), antibiotics (100 units/mL penicillin and 100 μg/mL streptomycin), and l-glutamine (2 mg/mL); these were passaged at a ratio of 1:3. Cells at passage 0–2 were used for subsequent experiments. DMEM, antibiotics, and l-glutamine were purchased from Invitrogen (Carlsbad, CA, USA). Fibroblastic mouse embryonic palatal mesenchyme cells that migrated from palatal shelves were physically removed after 24 h and were digested, subcultured, and passaged every 3–4 days. Cells at passage 2 were treated with 10 μM VO-Ohpic trihydrate (MedChemExpress, USA), an inhibitor of PTEN for 24 h before osteogenic differentiation.

### Transwell assay

Transwell assay was performed as previously described [27] with minor modifications. Briefly, passage 2 MEPM cells (5 × 10^5^ cells/mL) were trypsinized, washed, and resuspended in serum-free medium; 100 μL cells were incubated with 500 μL of 0.5% FBS medium or 0.5% FBS medium supplemented with PDGFAA (Sino Biological Inc., Beijing, China) at 37 °C for 24 h in a 5% CO_2_ humidified Transwell chamber. Subsequently, the Transwell inserts were fixed in 4% paraformaldehyde and then stained with Giemsa solution (Solarbio Life Sciences, Beijing, China). Filters of the inserts were then isolated with a scalpel and mounted. The migrated cells at the bottom of the Transwell chamber from three independent experiments were visualized under an Olympus BX51 microscope (Japan) and nine high-magnification fields were used for counting.

### Immunofluorescence

MEPM cells (3 × 10^5^ cells/well) were trypsinized (Invitrogen, Carlsbad, CA, USA) at passages 0 and 1, resuspended, and inoculated in six-well plates (Corning, USA), and subsequently subjected to immunofluorescence staining. Briefly, cells were incubated overnight at 4 °C with mouse mesodermal marker vimentin (1:100; ab92547; Abcam, USA), mouse pan-keratin (1:300; #4545T; Cell Signaling Technology), mouse ectodermal marker nestin IgG1 (1:300; ab11306; Abcam, USA), rabbit autophagosome protein [28], LC3A/B (1:100; #12741S; Cell Signaling Technology, USA), mouse neural crest marker HNK (1:100; sc81633; Santa Cruz Biotechnology, USA), monoclonal primary antibodies, PDGFRα (1:500; ab203491; Abcam, USA), Ephrin-B1(Efnb1) (1:10; sc515264; Santa Cruz Biotechnology, USA), Osr2 (1:10; sc81971; Santa Cruz Biotechnology, USA), and Meox2 (1:10; sc393516; Santa Cruz Biotechnology, USA) or PBS vehicle alone (negative control). Subsequently, these were incubated with secondary antibodies [Alexa Fluor 488-labeled goat anti-mouse or anti-rabbit IgG (1:300; A0428/A0423; Beyotime Inc., China), and/or Cy3-labeled goat anti-mouse IgG (1:500; A0521; Beyotime Inc., China)]. Cells were counterstained with 4′,6-diamidino-2-phenylindole (DAPI) (Beyotime Inc., China) at room temperature for 5 min. Vimentin-, Nestin-, LC3A/B-, HNK-1-, pan-keratin-, PDGFRα-, Ephrin-B1 (Efnb1)-, Osr2-, and Meox2-positive cells were assessed using an Olympus BX51 microscope (Japan).

### Goldner’s trichrome staining

Embryonic heads were fixed in 4% paraformaldehyde and subjected to stepwise ethanol dehydration, followed by paraffin embedding and sectioning. Deparaffinized sections (5 mm) were stained with Goldner’s trichrome kits (Solarbio Life Sciences, Beijing, China) according to the manufacturer’s instructions to examine the general morphology of the sections.

### Flow cytometry

The characteristics of MEPM cells were identified using the mouse mesenchymal stem cell (MSC) analysis kit (Cyagen Biosciences, Inc., Guangzhou, China). MEPM cells (3 × 10^6^ cells/mL) at passage 1 were harvested and resuspended in 100 μL buffer solution (1% PBS supplemented with 0.1% FBS) prior to incubation with control antibody (2 μL) (Armenian hamster IgG, rat IgG2b κisotype, or rat IgG2a κ isotype), or 2 μL of purified mouse CD29, CD44, CD117, Sca-1, CD31, CD34, or CD90.2 primary antibody, for 30 min on ice. The cells were subsequently incubated with 2 μL PE-conjugated goat anti-hamster IgG or PE-conjugated goat anti-rat IgG secondary antibody for 30 min on ice before analysis using a BD Accuri™ C6 flow cytometer (Becton-Dickinson, Franklin Lakes, NJ, USA).

### CCK-8 cell viability assay

MEPM cells (2 × 10^3^ cells/well) at passage 1 were seeded in 96-well plates (Corning, USA) and cultured in 100 μL DMEM, 10% FBS, and antibiotics. The absorbance at 450 nm was measured daily from day 1 to day 7 of culture after incubation with 10 μL CCK-8 solution (Beyotime Biotechnology, China) at 37 °C for an additional 1 h using an automatic multimode plate reader (EnSpire, Perkin Elmer Inc., USA).

### Multilineage differentiation

Multilineage differentiation of cells in passage 2 was performed as previously described [29–32] using an osteogenesis, chondrogenesis, and adipogenesis assay kit (Cyagen Biosciences, Inc.) according to the manufacturer’s instructions over a period of 2–4 weeks.

### Adipogenic differentiation

Cells in six-well plates that had achieved 80% confluence were treated with mouse bone mesenchymal stem cell (BMSC) adipogenesis-inducing medium (AIM) (Cyagen Biosciences, Inc., Suzhou, China) for 3 days. Subsequently, the medium was replaced and incubated in mouse BMSC adipogenesis-maintenance medium (AMM) (Cyagen Biosciences, Inc., Suzhou, China) for 24 h before switching back to AIM. After three rounds of media exchange, the cells were equilibrated in AMM for 1 week and adipogenic differentiation was assessed by staining with fresh Oil Red O solution (Solarbio Life Sciences, Beijing, China), as described elsewhere [33].

### Osteogenic differentiation

MEPM cells in six-well plates that had achieved 80–90% confluence were cultured in mouse BMSC osteogenic-inducing medium (Cyagen Biosciences, Inc., Suzhou, China) for 3 weeks with replacement of fresh culture medium every 3 days. Cells cultured in DMEM containing 10% fetal bovine serum, antibiotics, and l-glutamine was used as the control. Osteogenic differentiation was assessed by alkaline phosphatase (ALP) staining using an ALP analysis kit (Solarbio Life Sciences, Beijing, China) after 2 weeks of osteogenic induction. ALP activity was assessed as described elsewhere [34, 35]. Alizarin Red (AR) staining was then performed at the 3-week timepoint using AR solution (Solarbio Life Sciences, Beijing, China), as described elsewhere [36]. Briefly, MEPM cells were fixed with 4% paraformaldehyde for 30 min at 4 °C and then stained with 1% AR solution (1 mL, pH 4.2) at room temperature for 5 min before imaging using an Olympus BX51 microscope (Japan).

### Chondrogenic differentiation

Monolayer MEPM cells were cultured in mouse BMSC chondrogenic differentiation medium (Cyagen Biosciences, Inc., Suzhou, China) for 4 weeks with replacement of fresh culture medium every 3 days. Cells cultured in regular medium were used as the control. Chondrogenic differentiation was assessed by immunohistochemical staining for collagen type II (Col-II) (1:50; #TA311649S; OriGene Technologies, USA) under an Olympus BX51 light microscope (Japan).

### Real-time quantitative RT-PCR (qRT-PCR)

Total RNA was extracted with Trizol (Invitrogen, Thermo Scientific, USA) and reverse transcribed to cDNA using the PrimeScript RT reagent kit (TaKaRa, Japan). The RT-PCR reaction mix was prepared using the SYBR Premix Ex Taq kit (TaKaRa, Japan) and then real-time quantitative RT-PCR was performed using the Mx3000P qPCR system (Agilent Stratagene, Santa Clara, CA, USA). The qRT-PCR primer sequences are listed in Table 1.

**Table 1:** Primer sequences used for the relative quantification of the transcripts by qRT-PCR

Gene	Gene accession number	Forward primer	Reverse primer
Adiponectin	NM_009605.5	GCACTGGCAAGTTCTACTGCAA	GTAGGTGAAGAGAACGGCCTTGT
LPL	NM_008509.2	TGAGGATGGCAAGCAACACAACC	CATGAGCAGTTCTCCGATGTCCAC
ALP	NM_007432.2	CAACAGTGACAGCCACCAGGATC	GCTCACGCCGATGGTCTTGTAG
Cbfα-1	NM_001146038.2	AACAGCAGCAGCAGCAGCAG	GCACGGAGCACAGGAAGTTGG
COMP	NM_016685.2	ACAACTGCCGGTCCAAGAAGAATG	CGTATTCGGTCGCCATCTATGTCG
COL-II	NM_031163.3	GGTCCTCCTGGTCCTGGCATC	CGTGCTGTCTCAAGGTACTGTCTG

*LPL* lipoprotein lipase, *ALP* alkaline phosphatase, *Cbfα-1* core binding factor α1, *COMP* cartilage oligomeric matrix protein, *COL-II* collagen type II

### Western blot analysis

Cell lysates of undifferentiated and osteogenically differentiated MEPM cells were assessed after overnight incubation with LC3A/B (1:1000; #12741S), P62 (1:1000; #5114S), PTEN (1:1000; #9188 T), Akt (1:1000; #9272), mTOR (1:1000; #2972S), phospho-PTEN (1:1000; #9551 T), phospho-Akt (1:1000; #4060T), or phospho-mTOR (1:1000; #2971S) primary antibodies (Cell Signaling Technology, Danvers, MA, USA) at 4 °C and secondary antibodies (1:5000; #BE0101; #BE0102; Bioeasytech, Beijing; China) at room temperature for 1 h. Blots were developed using the enhanced chemiluminescence reagent (Beyotime Inc., China), and band intensities were analyzed using the ImageJ software (NIH, Bethesda, MD, USA).

### Transmission electron microscopy

Cells (5 × 10^4^–1 × 10^5^/condition) were centrifuged for 5 min at 4 °C at 800×*g* and then fixed on ice for 30 min in 0.1 M Na cacodylate, pH 7.4, containing 2% glutaraldehyde and 1% PFA before centrifugation at 1200×*g* for 10 min at 4 °C. Samples were then submitted to the Electron Microscopy Core Facility (ZHBY Biotech Co. Ltd., Nanchang, China) for standard transmission electron microscopy (TEM) analysis.

### Statistical analysis

Statistical analysis was performed using SPSS version 22.0 (IBM SPSS Inc., Chicago, IL, USA). All experiments were performed in triplicates. Comparisons were performed using Student’s *t* test or one-way ANOVA; *P* values < 0.05 were deemed statistically significant.

## Results

### Identification of migrated MEPM cells from palatal shelves

**Fig. 1:**
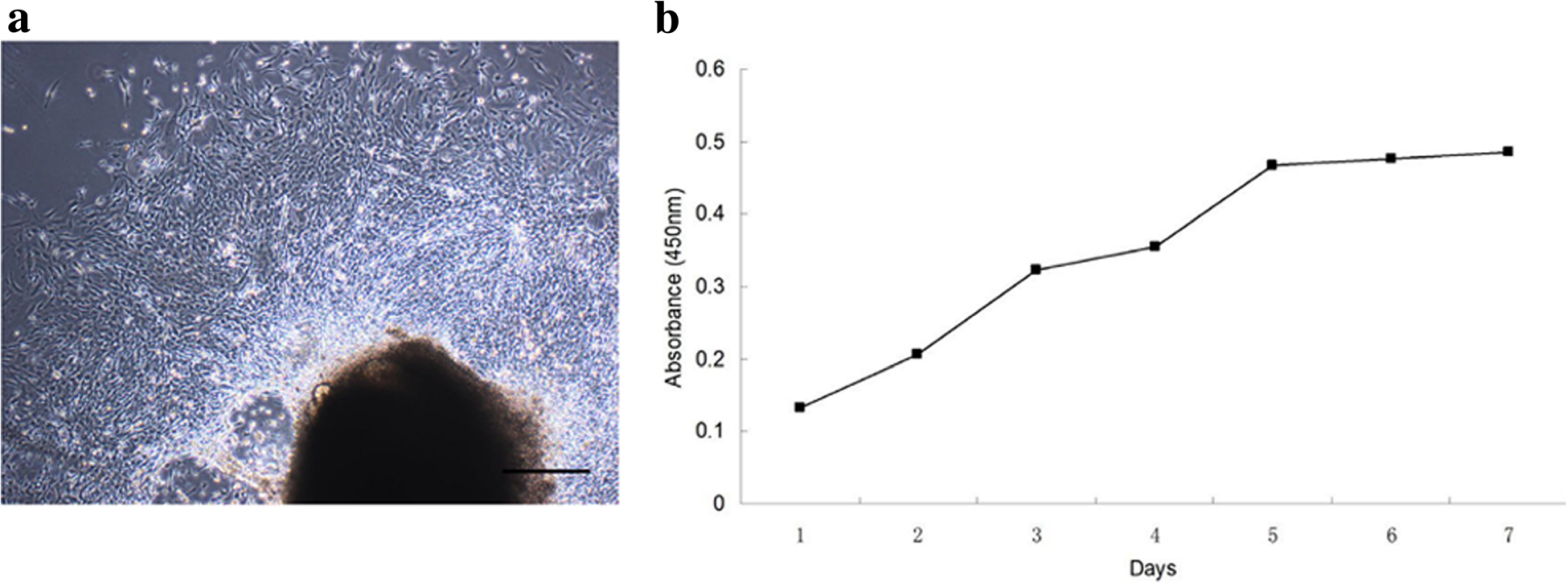
Identity and purity of MEPM cells. **a** MEPM cells emerging from palatal shelves. **b** CCK-8 assay profile showing MEPM cells proliferation at passage 1. Scale bar: 100 μm

**Fig. 2:**
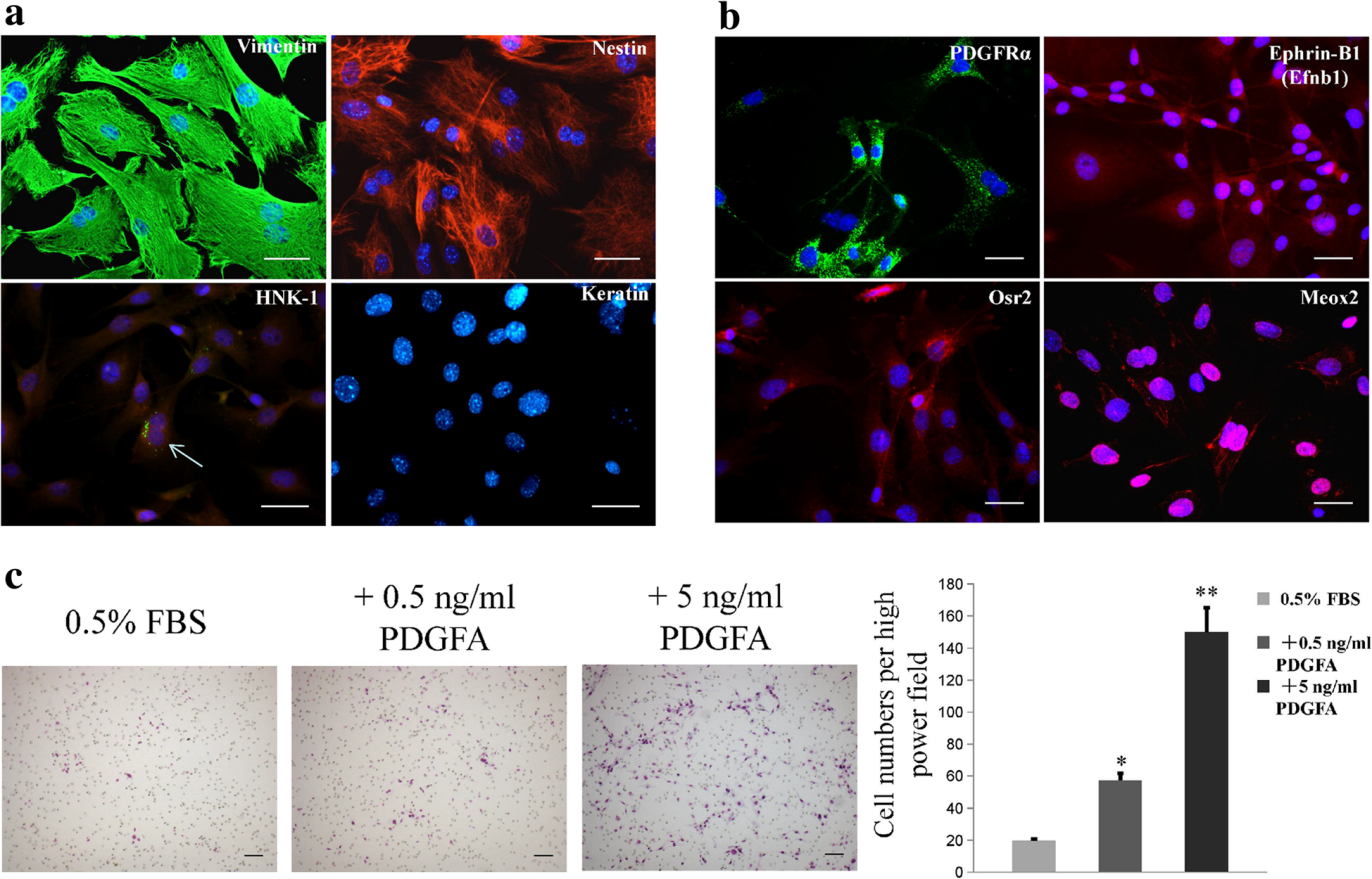
The properties of passage 1 MEPM cells. **a** Immunofluorescence staining of MEPM cells for vimentin (green), nestin (red), HNK-1 (green) (white arrow), and keratin (not stained). **b** Immunofluorescence staining of MEPM cells for PDGFRα (green), Efnb1 (red), Osr2 (red), and Meox2 (red). **c** Transwell assays showing that PDGFA (0.5 ng/mL and 5 ng/mL) stimulates chemotaxis of MEPMs. *N* = 9; *P < 0.05; **P < 0.01. Cell nuclei (blue) were counterstained with DAPI, and HNK-1 (red) in cytoplasm was stained with Evans blue dye. Scale bar: 20 μm

**Fig. 3:**
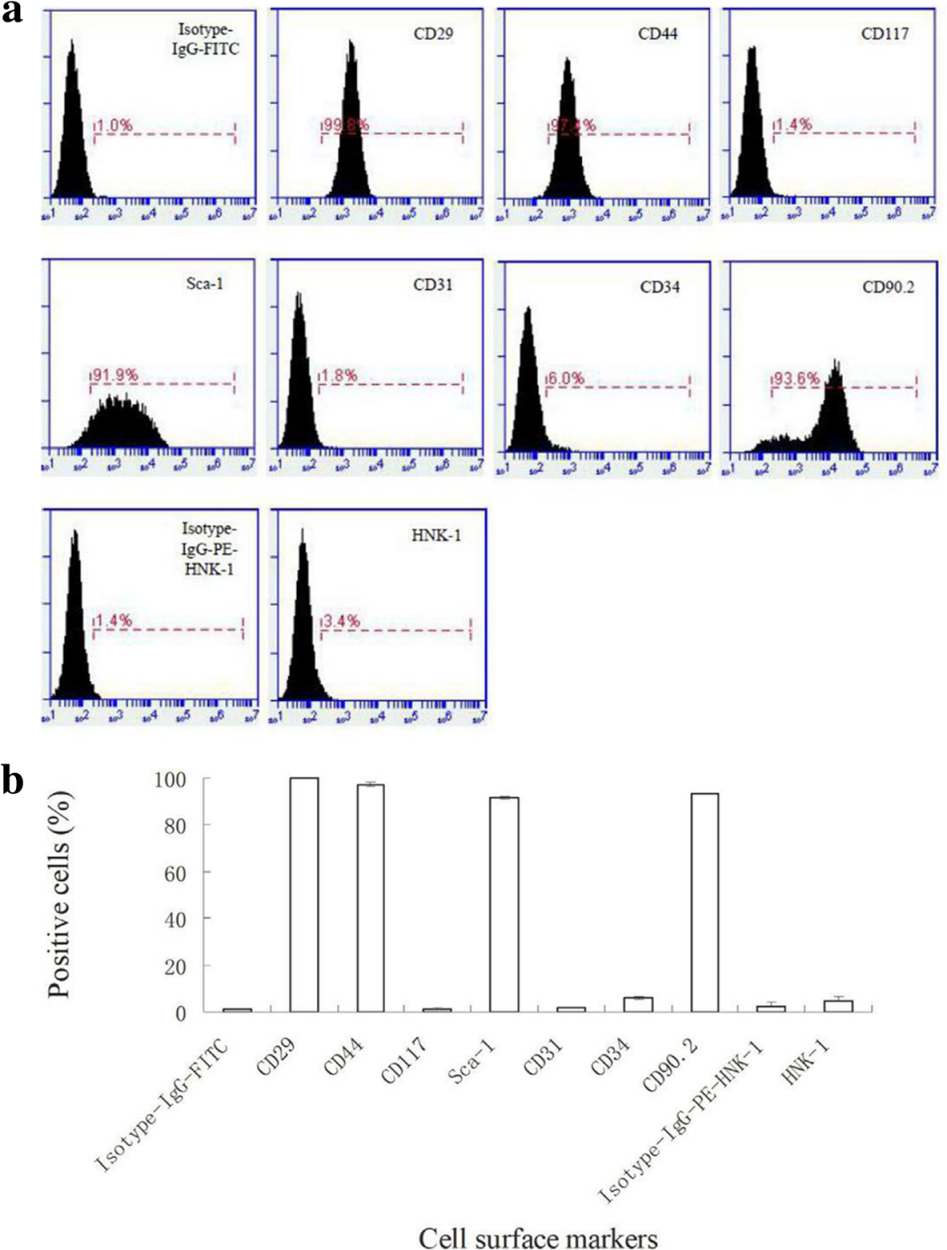
Immunophenotyping of MEPM cells. **a** Raw flow cytometry profiles of MEPM cells. **b** Semi-quantitative profiles from raw flow cytometry data showing cell surface marker expression levels in MEPM cells. Isotype gating using IgG-FITC and IgG-PE controls was performed to minimize non-specific signal

Fibroblastic MEPM cells migrated out of palatal shelves after 24 h (Fig. 1a) and exhibited positive staining for the mesodermal marker vimentin, ectodermal marker nestin, and neural crest marker HNK-1; however, the cells stained negative for keratin. HNK-1 staining indicated that the MEPM cells were derived from the cranial nerve crest. However, only 1% and 2% cells in the primary MEPM cell culture were keratin-positive and HNK-1-positive, respectively. The percentage of HNK-1-positive cells observed in this study is consistent with a previous report [37]. No keratin-stained cells were observed after passage 1; however, similar proportions of vimentin-, nestin-, and HNK-1-positive cells were observed after passage 1 (Fig. 2a), which suggests that cell passaging enabled MEPM cell specialization. MEPM cells were further confirmed by positive staining for the MEPM cell markers PDGFRα, Ephrin-B1(Efnb1), Osr2, and Meox2 (Fig. 2b). Transwell assay revealed that exposure to PDGFA stimulated chemotaxis of MEPM cell chemotaxis; this confirmed our immunofluorescence results that showed positive expression of PDGFRα on the isolated MEPM cells, which is consistent with the findings of a previous study [27] (Fig. 2c). Collectively, these findings confirmed that the cells isolated were indeed MEPM cells. Flow cytometry revealed that the cell surface marker expression on MEPM cells was similar to that of mouse bone mesenchymal stem cells (BMSC), such as CD29, CD44, CD90.2, Stro-1, and CD34 (Fig. 3a); the results showed high expression levels of CD29, CD44, CD90.2, and Stro-1, and low expression level of CD34. The high proliferation rate (Fig. 1b) and flow cytometry profiles obtained were consistent with that of ectomesenchymal stem cells.

### Stemness of MEPM cells

**Fig. 4:**
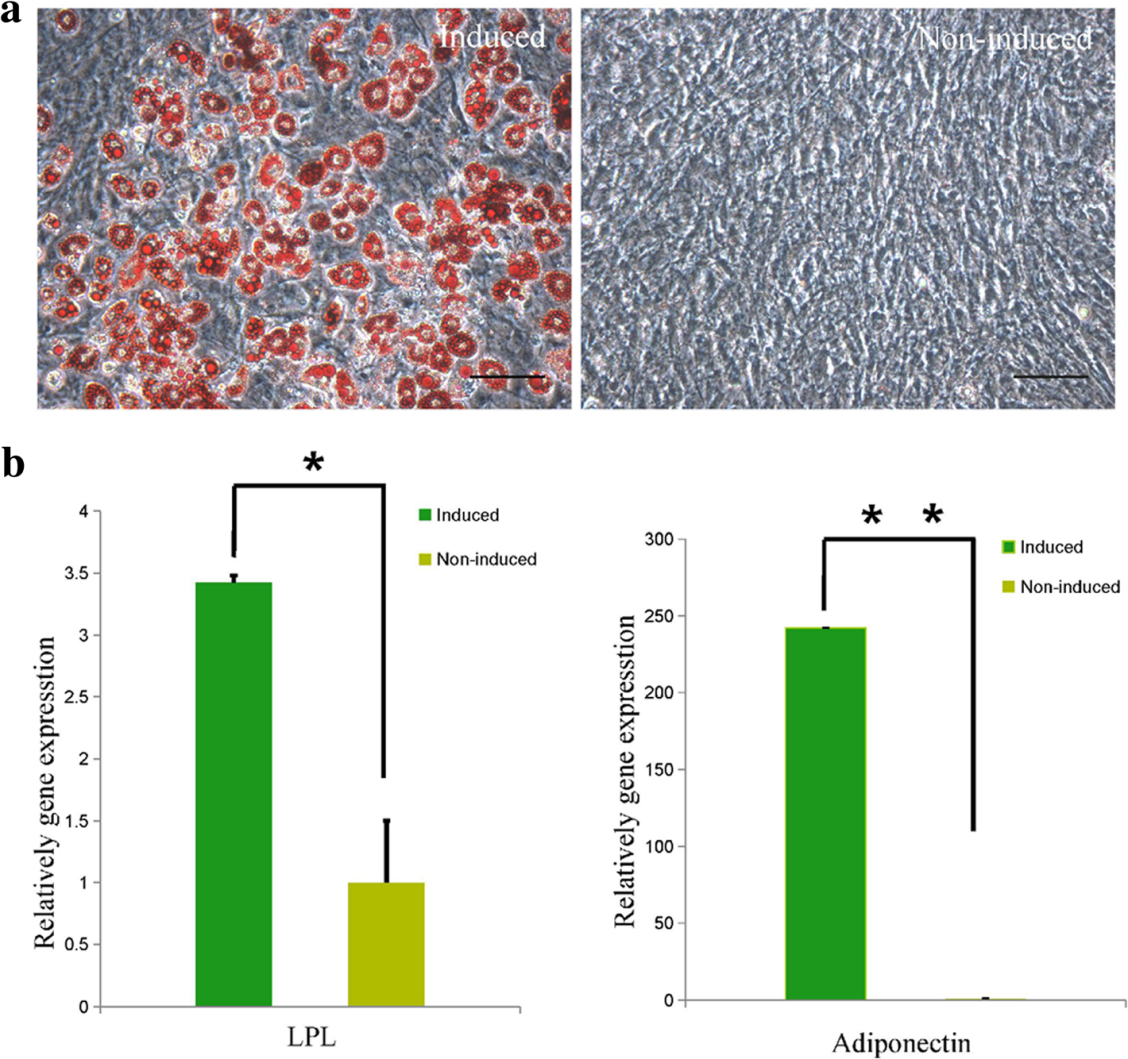
Adipogenic differentiation of MEPM cells. **a** Oil Red O-positively stained MEPM cells cultured in adipogenic-inducing (left panel) medium for 3 weeks and Oil Red O-negative MEPM cells cultured in regular culture medium (right panel). Scale bar: 50 μm. **b** qRT-PCR profile showing the expression levels of adiponectin and LPL in the induced and non-induced groups; *N* = 3; *P < 0.05; **P < 0.01

To confirm the stemness of MEPM cells, the adipogenic, osteogenic, and chondrogenic differentiation potential were determined. After 3-week culture in adipogenic medium, the percentage of Oil Red O-positive MEPM cells was approximately 15% as against very few Oil Red O-positive cells in the control group (Fig. 4a); this indicated the potential of MEPM cells for adipogenic differentiation. The results were further confirmed by qRT-PCR analysis (Fig. 4b). The induced group showed significantly higher expression levels of lipoprotein lipase (LPL) and adiponectin (adipogenic-related genes).

**Fig. 5:**
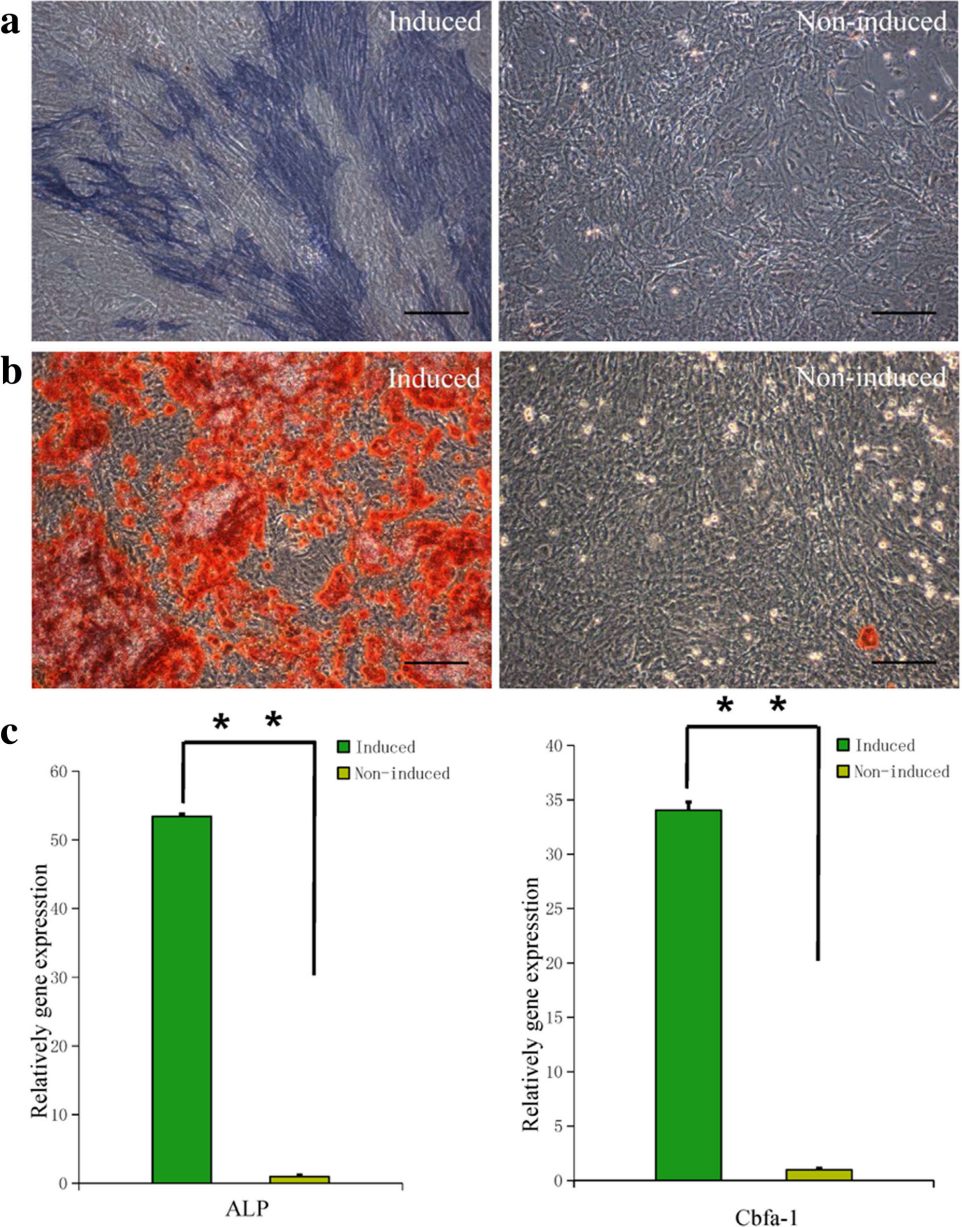
Osteogenic differentiation of MEPM cells. **a** ALP staining of induced (left panel) and non-induced (right panel) MEPM cells after 2 weeks of osteogenic induction; scale bar: 50 μm. **b** AR staining of induced (left panel) and non-induced (right panel) MEPM cells after 3 weeks of osteogenic induction (scale bar: 50 μm). **c** qRT-PCR profile showing ALP and Cbfα-1 expression levels in the induced and non-induced groups; *N* = 3; **P < 0.01

We observed over 20% ALP-positive cells after 2 weeks of osteogenic induction (Fig. 5a); in addition, AR staining revealed intracellular calcium deposition. Approximately 90% cells exhibited mineralized nodules after 3 weeks of osteogenic induction (Fig. 5b). Extensive mineralization indicated that MEPM cells possessed high osteogenic differentiation potential. The ALP and AR staining results were verified by qRT-PCR analysis (Fig. 5c); there was a significant increase in the expression of osteogenic-related genes ALP and core binding factor α1 (Cbfα-1). These findings confirmed the osteogenic potential of MEPM cells.

**Fig. 6:**
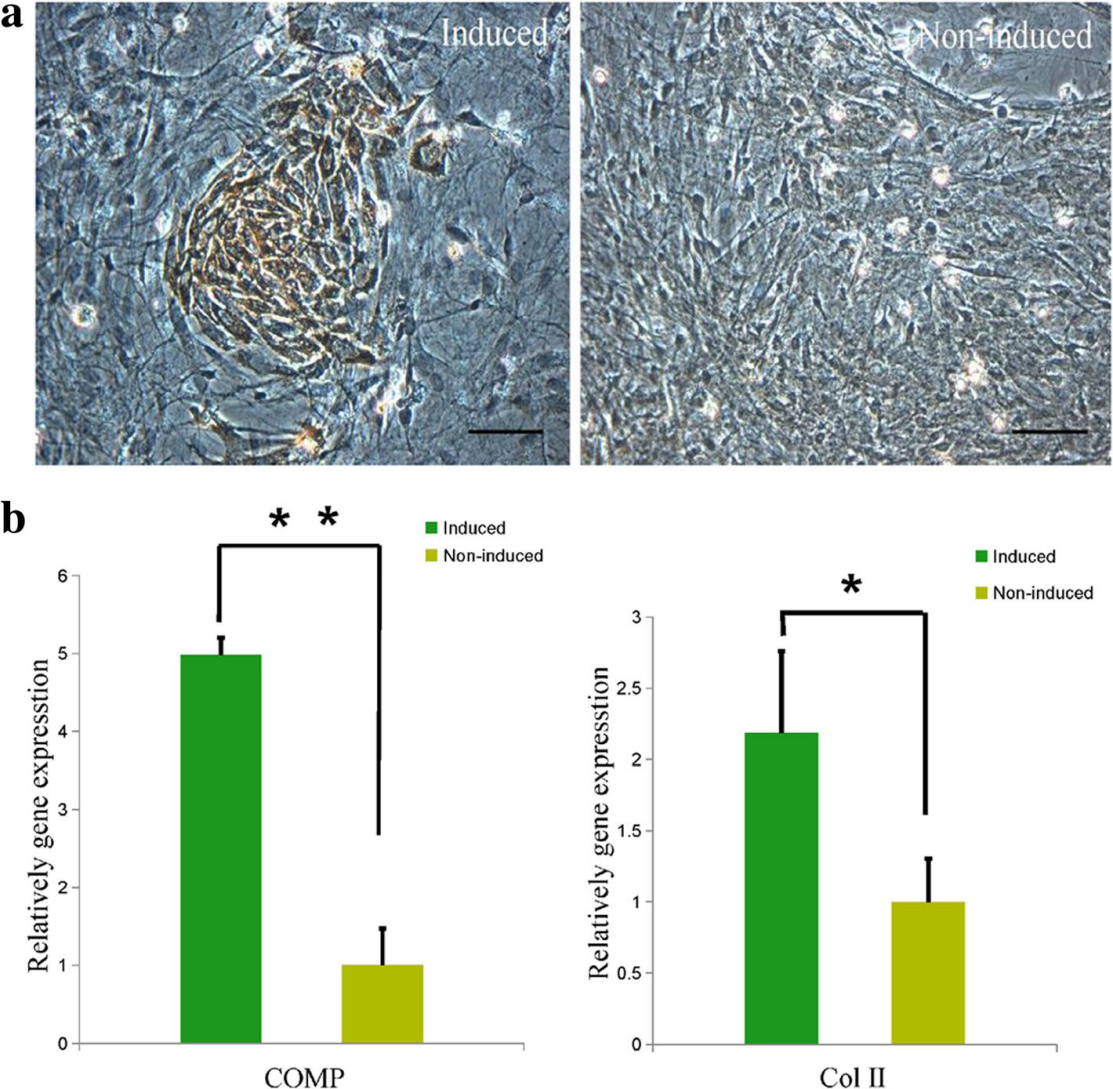
Chondrogenic differentiation of MEPM cells. **a** Immunohistochemical staining of Col-II in induced (left panel) and non-induced (right panel) groups; scale bar: 50 μm. **b** qRT-PCR profile showing COMP and Col-II expression levels in the induced and non-induced groups; *N* = 3; *P < 0.05; **P < 0.01

On immunohistochemical staining, approximately 2–3.5% cells stained positive for Col-II (Fig. 6a), which indicated the chondrogenic differentiation potential of MEPM cells. In addition, qRT-PCR analysis showed high expressions of Col-II and cartilage oligomeric matrix protein (COMP) in the induced cells (Fig. 6b), which also demonstrated the potential of MEPM cells to differentiate into chondrogenic cells.

### Autophagy maintains MEPM stemness

**Fig. 7:**
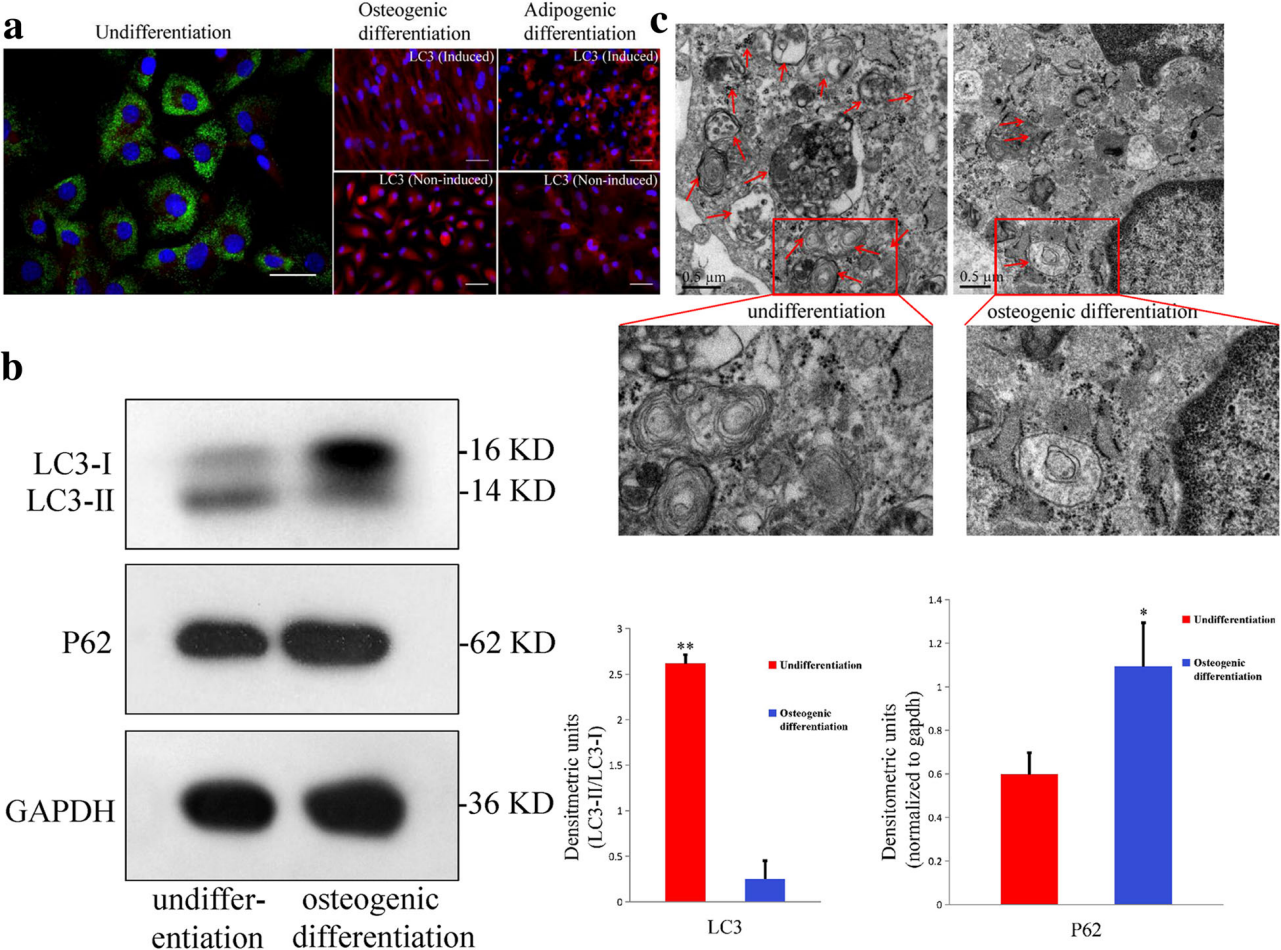
Characteristics of autophagy in MEPM cells. **a** LC3-II immunofluorescence staining before differentiation and after osteogenic and adipogenic differentiation. Cell nuclei (blue) were counterstained with DAPI; cytoplasm (red) was stained with Evans blue; scale bar: 20 μm. **b** Western blot showing expression levels of LC3-I/II and p62 proteins before and after osteogenic differentiation; *N* = 3; *P < 0.05; **P < 0.01. **c** TEM image showing large amount of autophagosomes (red arrows) before osteogenic differentiation, which decreased sharply after osteogenic differentiation

**Fig. 8:**
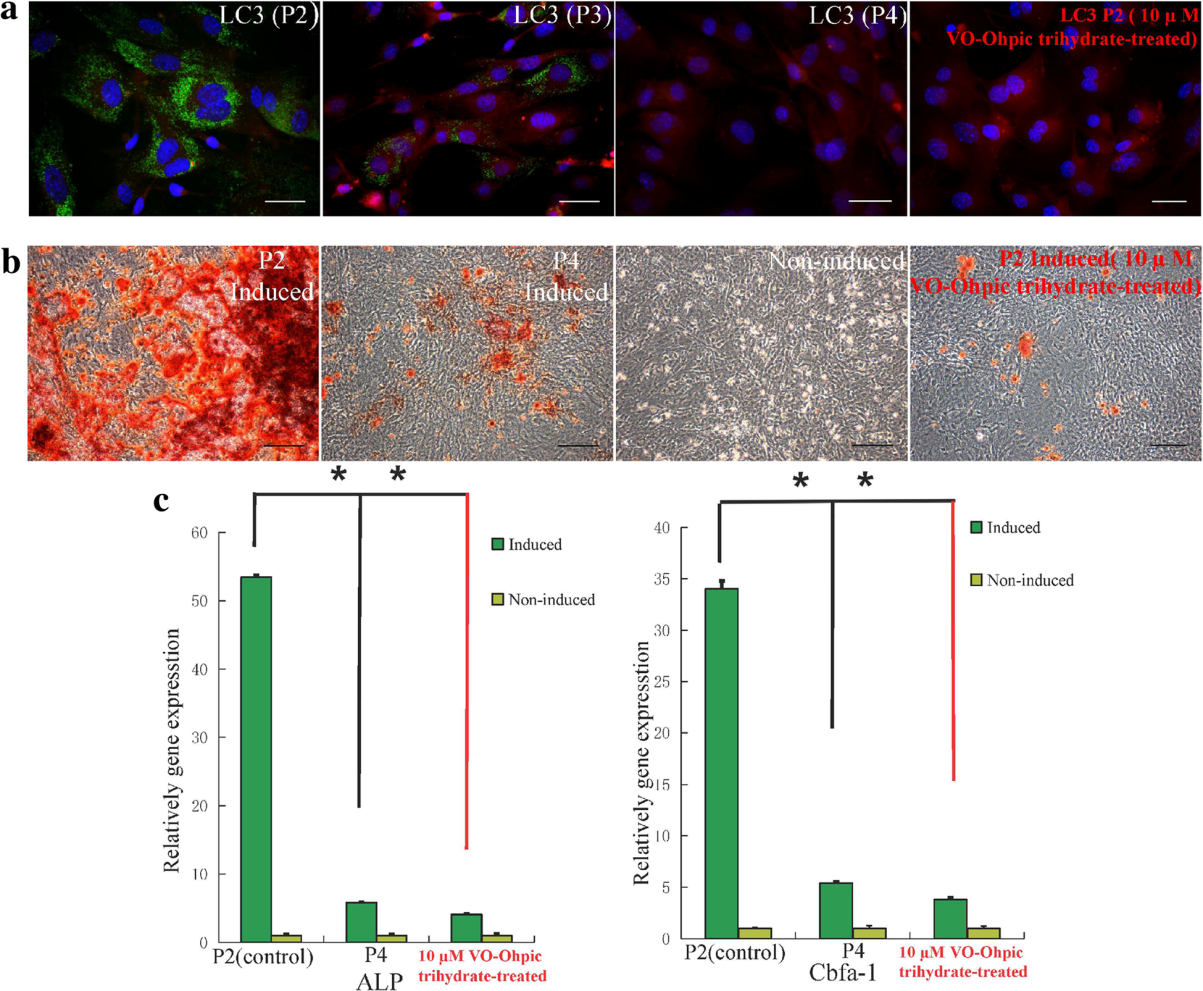
Characteristics of MEPM cells at passage 4. **a** LC3-II immunofluorescence staining; scale bar: 20 μm. **b** AR staining of induced, non-induced, and VO-OHpic trihydrate-treated MEPM cells after 3 weeks of osteogenic induction; scale bar: 50 μm. **c** qRT-PCR profile showing ALP and Cbfα-1 expression levels in the induced, non-induced, and VO-OHpic trihydrate-treated groups; *N* = 3; **P < 0.01

The role of autophagy in stem cell survival and the properties of stemness have been extensively studied in recent years [21–23]. MSCs are known to contain higher levels of autophagosomes than those in differentiated cells [2, 21, 28, 38]. To determine whether undifferentiated MEPM cells exhibit stemness like MSCs, we investigated their autophagic activity using LC3 type II (LC3-II) immunofluorescence staining, Western blotting, and TEM and compared that with culture in differentiation medium. A large number of autophagosomes were observed in undifferentiated MEPM cells, which were significantly reduced after osteogenic and adipogenic differentiation (Fig. 7a, c). Significantly decreased number of autophagosomes was observed (Fig. 7a–c) in the control groups after 3-week culture. The LC3-I/LC3-II ratio and the autophagy adaptor protein p62 level were significantly increased after osteogenic differentiation. Moreover, the number of autophagosomes decreased with passage and all the autophagosomes had disappeared by passage 4 (Fig. 8a). Next, the osteogenic differentiation potential of MEPM cells at passage 4 was determined to ascertain the role of autophagy in maintaining stemness of MEPM cells. After 3 weeks of osteogenic induction, only about 5% MEPM cells at passage 4 exhibited mineralized nodules (Fig. 8b), which was much lower than that at passage 2. This observation was confirmed by qRT-PCR analysis. In addition, there was downregulation of osteogenic differentiation-related genes, ALP and Cbfα-1, in MEPM cells at passage 4 compared to that at passage 2 (Fig. 8c); this indicated that the reduction in autophagy level corresponded to the loss of MEPM stemness.

### MEPM cells maintain stemness through PTEN-Akt-mTOR autophagy signaling

**Fig. 9:**
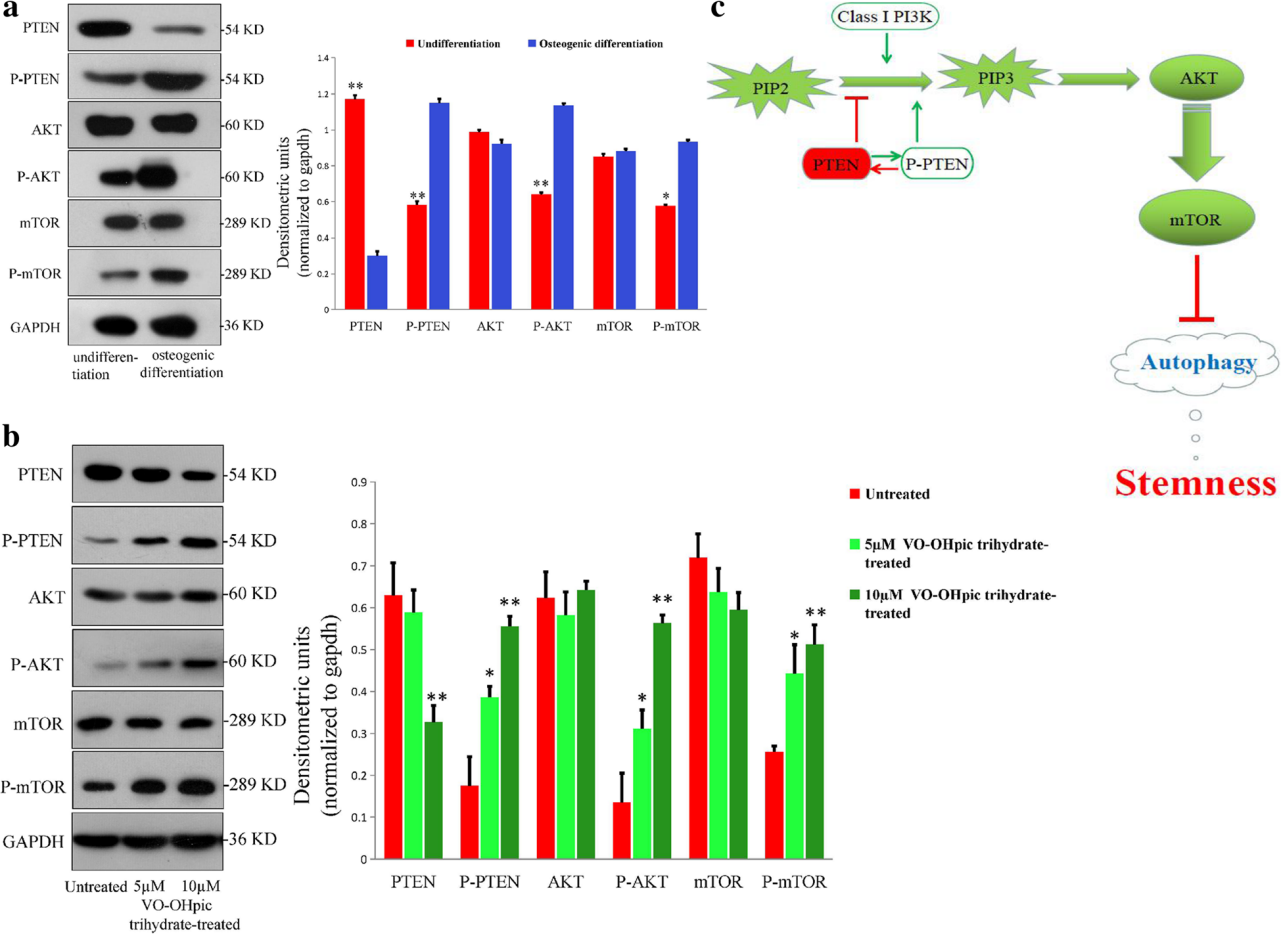
PTEN-Akt-mTOR autophagic signaling. **a** Western blot showing the levels of phosphorylated and non-phosphorylated PTEN, AKT, and mTOR proteins before and after osteogenic differentiation. **b** Western blot showing the levels of phosphorylated and non-phosphorylated PTEN, AKT, and mTOR proteins before and after VO-OHpic trihydrate treatment. **c** Schematic illustration of the autophagic pathway. *N* = 3; *P < 0.05; **P < 0.01

PI3K/Akt/mTOR signaling negatively regulates autophagy [39] while the tumor suppressor PTEN activates autophagy via suppression of PI3K/Akt/mTOR signaling [40]. We performed Western blot analysis of pan- and phosphorylated PTEN, Akt, and mTOR to determine the role of PTEN-Akt-mTOR signaling in maintaining MEPM stemness. Increased PTEN phosphorylation (which is synonymous with PTEN inactivation) and reduced total PTEN protein expression were observed after osteogenic differentiation (Fig. 9a). In addition, we observed significantly increased phosphorylation of the autophagic inhibitors, AKT and mTOR; however, there was no change in the total expression of AKT and mTOR proteins. Undifferentiated MEPM cells exhibited low phospho-PTEN levels, high total PTEN expression, and low phosphorylated AKT and mTOR levels, indicating that MEPM cells maintained stemness through PTEN-Akt-mTOR autophagy signaling. Western blot analysis of MEPM cells treated with the PTEN inhibitor VO-OHpic trihydrate showed successful downregulation of PTEN protein level, accompanied with concomitant increase in phosphorylated AKT and mTOR levels but no change in AKT and mTOR protein levels (Fig. 9b). Moreover, LC3 type II (LC3-II) immunofluorescence staining of MEPM cells exposed to VO-Ohpic trihydrate revealed the disappearance of autophagosomes (Fig. 8a). Furthermore, the osteogenic differentiation potential of MEPM cells was lost as evident from ARS staining and qRT-PCR of ALP and Cbfα-1 (Fig. 8b, c). Taken together, these results showed that MEPM cells maintain stemness through PTEN-Akt-mTOR autophagic signaling (Fig. 9c).

## Discussion

Self-renewal ability and pluripotency are key attributes of stem cells that enable them to function in multiple stages of development. Progenitor stem cells are involved in early embryonal development and subsequently become specialized in the later developmental stages by differentiating into restricted cell types [41]. In this study, we investigated the stemness of MEPM cells in palatal shelves to better understand the development of palatal shelves. On immunofluorescence examination, MEPM cells exhibited weak HNK-1 staining and positive expression of MSC surface markers, which indicates their similarity to MSCs. In addition, MEPM cells exhibited the ability for adipogenic, osteogenic, and chondrocytic differentiation, which suggests that these are ectomesenchymal stem cells. Western blot analysis of the protein levels of the factors in the PTEN/Akt/mTOR autophagic pathway revealed their essential role in maintaining MEPM stemness.

Although MEPM cells are derived from CNCCs, only 2% of the cells expressed the neural crest marker HNK-1. This may be due to the admixture comprising several types of CNCCs, or to differentiation of a significant portion of CNCCs in the MEPM cell culture which may have prevented their detection by immunostaining; alternatively, we may have used a less optimal culture medium instead of serum-free medium for CNCC culture, which may have affected HNK-1 staining. Nevertheless, our results are consistent with those of Gazarian et al. [37].

**Fig. 10:**
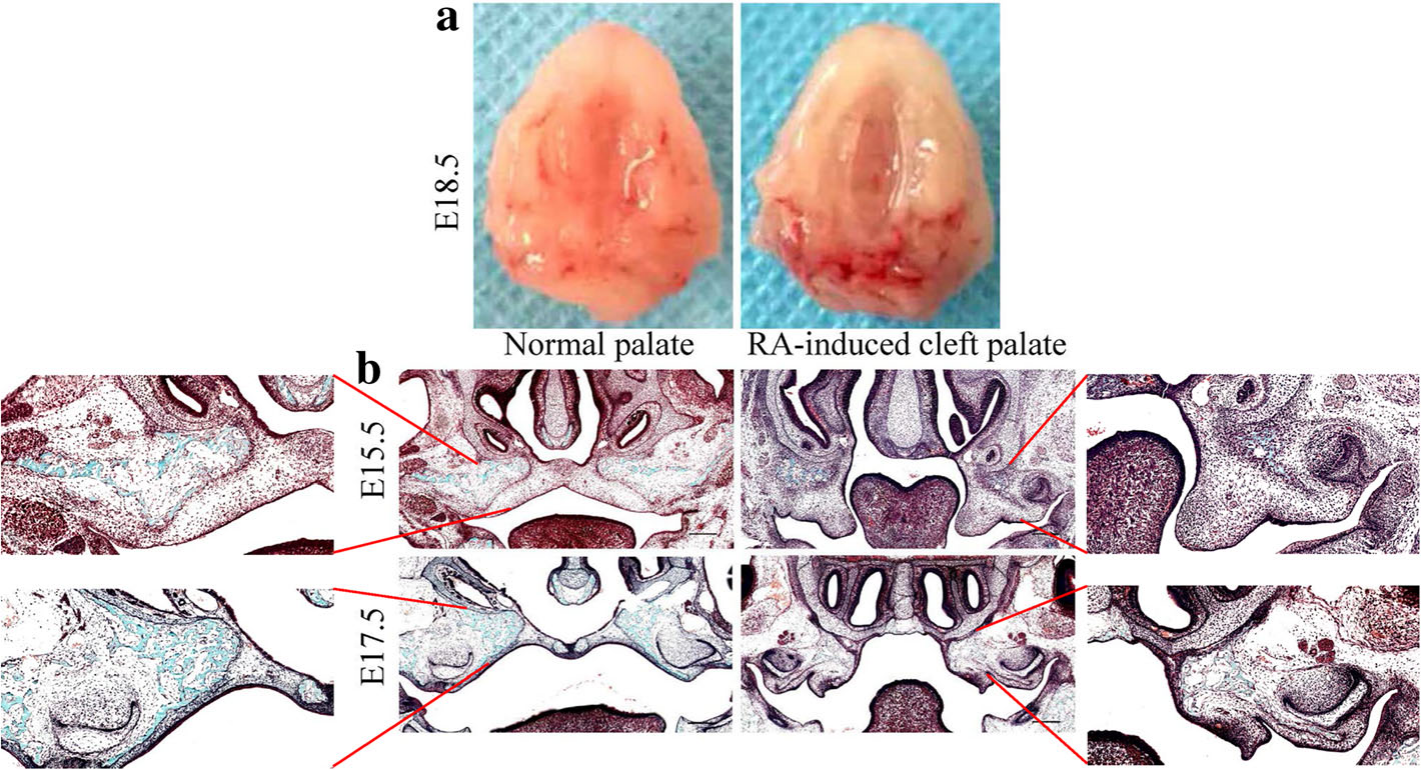
RA-induced cleft palate in Kunming mice. **a** Comparison of general palatal morphology between the normal group and RA-induced group. **b** Goldner’s trichrome staining of frontal sections of palatal regions at E15.5 and E17.5 in normal palate and RA-induced cleft palate. Mineralized bones are stained green; osteoid are stained orange/red; chondrocyte are stained purple; nuclei are stained blue/gray; and the cytoplasm is stained red/pink. Scale bar: 200 μm

During palate development, the ossified hard palate forms the anterior two thirds of the palate while the soft palate forms from the posterior one third of the palate [42]. In the multilineage differentiation experiments, approximately 90% of MEPM cells were found to contain mineralized nodules after 3 weeks of osteogenic induction, which suggests that MEPM cells possess stronger potential for osteogenic differentiation as compared to that for adipogenic and chondrogenic differentiation. Moreover, a significant proportion of MEPM subpopulations tended towards osteogenic differentiation during palatal development. The palatal shelves switch from vertical to horizontal growth towards each other above the dorsum of the tongue at around E14.0; this indicates that osteogenic differentiation and MEPM cell proliferation are synergistic processes and that osteogenic differentiation is crucial for palatal shelf elevation and subsequent MEPM cell proliferation to enable palate formation. These findings suggest that disruption of MEPM stemness and osteogenic differentiation likely play a role in the pathogenesis of cleft palate. Indeed, abnormal osteogenic differentiation [43–45] and abnormal osteogenic signaling prior to the elevation of palatal shelves [46] were shown to contribute to the development of cleft palate. Moreover, we found that disruption of MEPM osteogenic differentiation upon RA induction may lead to cleft palate formation (Fig. 10a) as we observed significantly fewer regions of mineralized bone in the RA-induced cleft palate than in normal palate (Fig. 10b).

A high autophagy level is crucial for stem cell survival under harsh conditions [20]. In this study, we observed a high density of autophagosomes in primary MEPM cells and these disappeared after adipogenic, osteogenic, or chondrogenic differentiation; this suggests the occurrence of sustained autophagy in undifferentiated MEPM cells prior to lysosomal degradation. In this way, autophagy enables the rapid generation and continuous supply of amino acid building blocks to support MEPM differentiation. Thus, the autophagy level may serve as an indicator of MEPM stemness.

The PI3K/Akt/mTOR pathway negatively regulates autophagy by modulating cell growth, motility, protein synthesis, cell metabolism, cell survival, and cell death in response to various stimuli [47, 48]. This pathway is in turn regulated by PTEN wherein active unphosphorylated PTEN suppresses PI3K and Akt [49]. Here, reduced autophagy level was noted with repeated MEPM passaging and osteogenic differentiation induced PTEN phosphorylation, which in turn activated PI3K/Akt/mTOR signaling. These findings suggest that undifferentiated MEPM cells likely maintain high basal levels of autophagy through PTEN/AKT/mTOR signaling to maintain stemness and disruption in autophagy signaling, which led to cleft palate formation.

In conclusion, we demonstrate that MEPM cells are ectomesenchymal stem cells with strong osteogenic differentiation potential and that their stemness is regulated by PTEN/AKT/mTOR autophagic signaling. Activation of the PTEN/AKT/mTOR pathway may prevent the formation of cleft palate. Our findings provide new insights into the underlying mechanism of cleft palate formation and may help identify new candidate markers for prenatal screening for cleft palate, and new targets for its diagnosis and treatment.

## Conclusions

Our findings suggest that MEPM cells are ectomesenchymal stem cells with a strong osteogenic differentiation potential and that maintenance of their stemness via PTEN/AKT/mTOR autophagic signaling prevents cleft palate development.

## Data Availability

The authors declare that all the data supporting the findings of this study are available within the article and that no data sharing is applicable to this article.

## Abbreviations

AIM: Adipogenesis-inducing medium
ALP: Alkaline phosphatase
AMM: Adipogenesis-maintenance medium
AR: Alizarin Red
BMSC: Bone mesenchymal stem cell
Cbfα-1: Core binding factor α1
CNCCs: Cranial neural crest cells
COMP: Cartilage oligomeric matrix protein
DAPI: 4′,6-Diamidino-2-phenylindole
DMEM: Dulbecco’s modified Eagle’s medium
E: Embryonic day
FBS: Fetal bovine serum
LC3-II: LC3 type II
LPL: Lipoprotein lipase
MEE: Medial edge epithelial
MEPM: Mouse embryonic palatal mesenchyme
MES: Medial epithelial seam
MSC: Mesenchymal stem cell
Osr2: Protein odd-skipped-related 2
PDGF: Platelet-derived growth factor
RA: Retinoic acid
TEM: Transmission electron microscopy
